# Modified Constraint-Induced Movement Therapy for Improving Balance and Gait in a Case of Ganglio-Capsular Infarct: A Single-Case Study

**DOI:** 10.7759/cureus.55420

**Published:** 2024-03-03

**Authors:** Nitika Chavan, Raghumahanti Raghuveer

**Affiliations:** 1 Neurophysiotherapy, Ravi Nair Physiotherapy College, Datta Meghe Institute of Higher Education and Research, Wardha, IND

**Keywords:** modified constraint-induced movement therapy, neurophysiotherapy, stroke, rehabilitation, ganglio-capsular infarct

## Abstract

The ganglio-capsular region consists of the basal ganglia nuclei (caudate nucleus and lentiform nucleus), thalamus, and internal capsule. A disorder of the ganglio-capsular region typically presents with movement disturbance and cognitive impairment. This report presents the case of a 52-year-old male who was diagnosed with acute non-hemorrhagic infarct in the right parietal-occipital-temporal region predominantly involving the cortex and in the right ganglio-capsular region. The patient exhibited typical symptoms, which include impaired reflexes, decreased strength, reduced range of motion, and tone abnormalities. Targeted early physiotherapy intervention (TERI) was initiated from the bedside in the intensive care unit (ICU). Modified constraint-induced movement therapy (mCIMT) along with conventional therapy was selected as the rehabilitation approach for the case as it deals with "forced use" of the affected extremities, which addresses "learned non-use." The case was managed for a duration of six weeks, in which clinical outcomes, including the Berg Balance Scale (BBS), 10-meter walk test (10MWT), functional reach test (FRT), dynamic gait index (DGI), trunk impairment scale (TIS), and fall efficacy test (FET), reported crucial changes in balance, strength, coordination, and tone, which improved the quality of life of the patient.

## Introduction

There are three types of brain infarcts: transcerebral, subcortical, and cortical. Infarcts isolated in the ganglio-capsular area, corona radiata, and centrum semiovale are referred to as subcortical infarcts. Ganglio-capsular infarcts are the most common form of infarcts, which occur due to long-standing uncontrolled hypertension. The incidence is 80-120 per 100,000 among Asians [1]. The simultaneous occlusion of multiple nearby lenticulostriate arteries results in a ganglio-capsular infarct. The proximal portion of the middle cerebral artery (MCA) gives rise to deep perforators, known as lenticulostriate arteries. These branches with profound perforations supply the head and body [2]. A ganglio-capsular infarct, also known as stroke, is a severe neurological condition that ranks third in terms of mortality (after cancer and heart disease) [3].

Movement abnormalities in the lower extremities, such as paralysis, weakness, and atypical synergistic movement patterns, are frequently observed in the opposite direction of the infarct. Post-stroke impairment in the lower limb significantly alters gait parameters, causing loss of balance, instability in walking gait, and slower gait, which may hamper walking ability [4]. Stroke is the most often treated disability by physical therapists. For many stroke patients, regaining walking ability is a main goal and a priority of rehabilitation [5]. The primary goal of stroke rehabilitation is to regain autonomy [6]. For stroke patients, there are various physiotherapy approaches, including constraint-induced movement therapy (CIMT), modified constraint-induced movement therapy (mCIMT), motor relearning program, virtual reality therapy, robot-assisted training, and proprioceptive neuromuscular facilitation. Every strategy follows its own set of guidelines to restore voluntary control and movement [7].

The original CIMT entails using a safety mitt that is used to restrain the patient's less compromised extremity. CIMT is a rigorous treatment that can be challenging to implement since patients can become fatigued easily [8]. Therapists have recognized the need for time and the challenges involved in a six-hour program. As an alternative to the intensive nature of CIMT, the mCIMT protocol was created, requiring reduced time utilization constraints over a longer intervention period [9]. mCIMT consists of three fundamental components: practice-intensive therapy in the paretic limb, and the use of repetitive and non-paretic limbs is constrained. mCIMT has recently been applied to the lower leg of the paretic in order to improve neurological function and prevent "learned non-use" [7]. The study aims to see the effect of targeted early rehabilitation intervention using mCIMT in enhancing the quality of life of patients.

## Case presentation

Patient information

A 52-year-old male was brought to a tertiary hospital with complaints of lack of movement on the left half of his body (both trunk and extremities). He had trouble performing activities of daily living (ADLs) and had difficulty walking or standing after his stroke. He was promptly admitted to the tertiary hospital by his brother. A magnetic resonance imaging (MRI) of the brain with angiography was performed. Furthermore, he was referred to neurophysiotherapy.

Clinical findings

**Figure 1:**
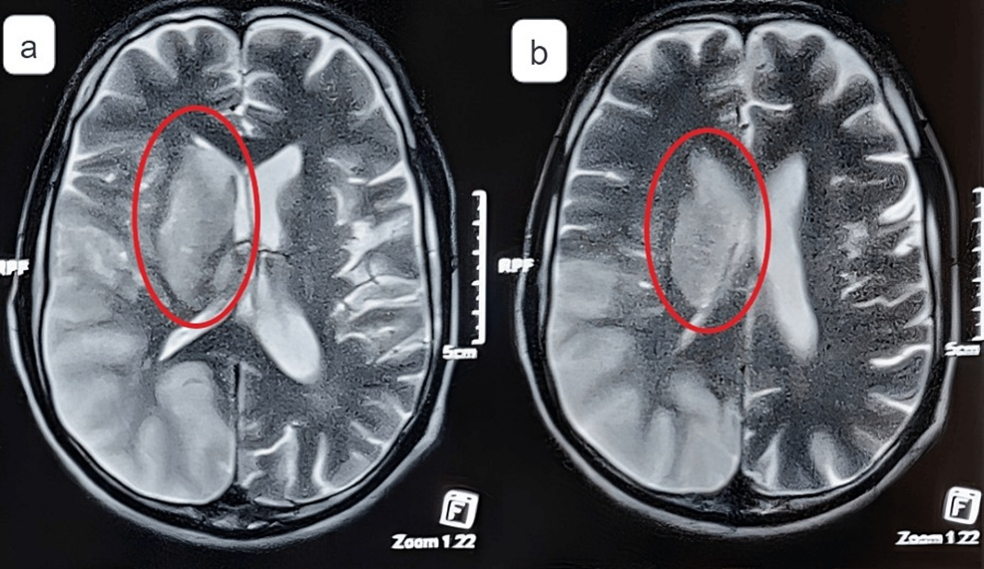
MRI reveals an acute non-hemorrhagic infarct noted in the right parietal-temporal-occipital region, predominantly involving the cortex and right ganglio-capsular region. The right lateral ventricle was observed to have effaced due to the mass effect and midline shift of 4 mm toward the left side. A: acute non-hemorrhagic infarct involving the cortex and right ganglio-capsular region, B: acute non-hemorrhagic infarct involving the right ganglio-capsular region MRI: magnetic resonance imaging

**Figure 2:**
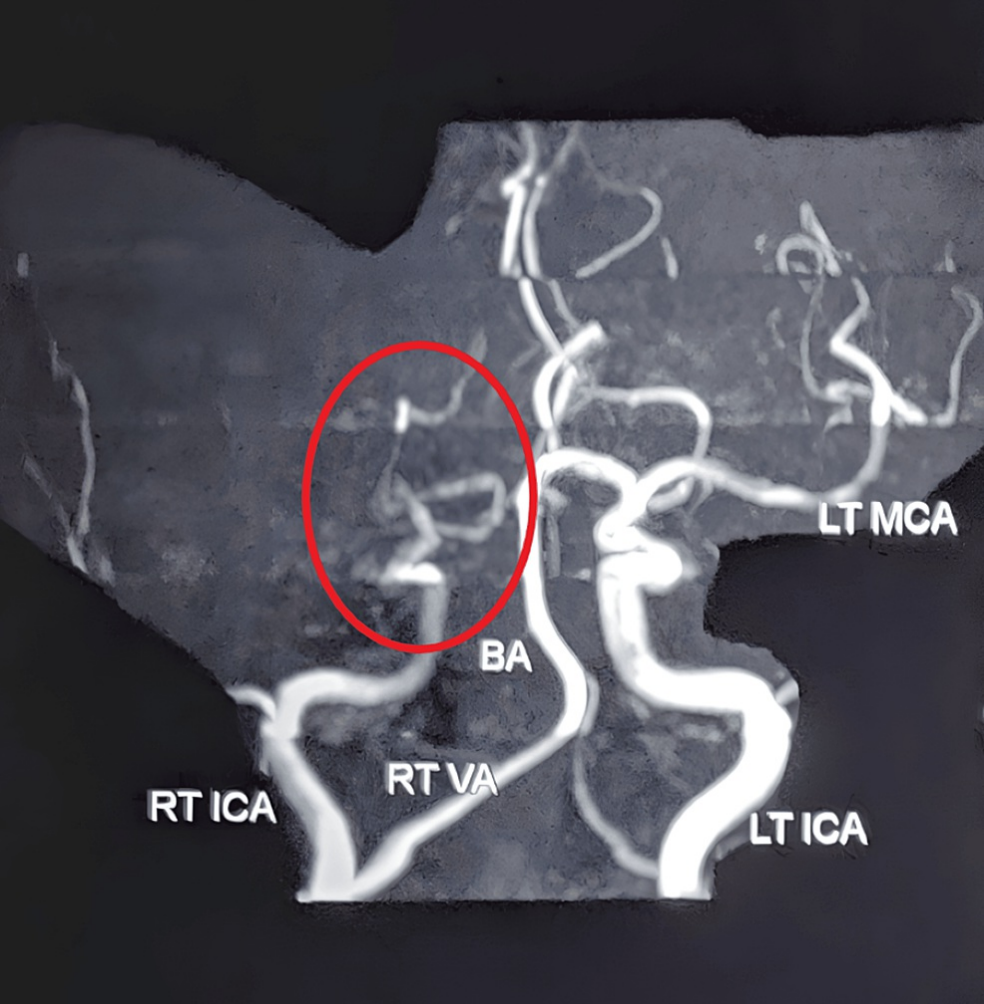
MR angiography study reveals thrombotic occlusion noted in the entire right middle cerebral artery. MR: magnetic resonance

After receiving written consent, a full assessment was performed. All superficial and deep sensations were intact. In the pre-rehabilitation assessment, the tone was hypotonic (shown in Table 1). The superficial and cerebral reflexes of the patient were intact. Deep reflexes were absent in pre-rehabilitation (shown in Table 2). There were no cognitive deficits, such as agnosia, apraxia, or hemispatial neglect. Voluntary control grading was assessed pre and post rehabilitation (shown in Table 3). The outcome measures used were the Berg Balance Scale, dynamic gait index, trunk impairment scale, functional reach test, 10-meter walk test, and fall efficacy test (shown in Table 4). An investigation was done with an MRI and brain angiography (shown in Figure 1 and Figure 2, respectively).

**Table 1:** Tone assessment (tone grading scale) 1+ = hypotonia, 2+ = normal

	Pre-rehabilitation left	Post rehabilitation left
Shoulder flexors	1+	2+
Shoulder extensors	1+	2+
Shoulder abductors	1+	2+
Shoulder adductors	1+	2+
Elbow flexors	2+	2+
Elbow extensors	2+	2+
Wrist flexors	2+	2+
Wrist extensors	2+	2+
Hip flexors	1+	2+
Hip extensors	1+	2+
Knee flexors	1+	2+
Knee extensors	1+	2+
Ankle dorsiflexors	2+	2+
Ankle plantarflexors	2+	2+

**Table 2:** Reflexes 0= absent, += diminished, ++ = normal

	Pre-rehabilitation left	Post rehabilitation left
Biceps jerk	+	++
Triceps jerk	Absent	++
Supinator jerk	+	++
Knee jerk	Absent	++
Ankle jerk	Absent	++
Plantar response	++	++

**Table 3:** Voluntary control grading (VCG)

	Pre-rehabilitation left side	Post-rehabilitation left side
Shoulder flexors	2	5
Shoulder extensors	2	5
Shoulder abductors	2	5
Shoulder adductors	2	6
Elbow flexors	2	6
Elbow extensors	2	6
Wrist flexors	2	6
Wrist extensors	2	6
Hip flexors	1	6
Hip extensors	1	5
Knee flexors	1	5
Knee extensors	1	5
Ankle dorsiflexors	1	6
Ankle plantarflexors	1	6

**Table 4:** Upper extremity, pelvic position, and lower-extremity stretching exercises

Muscle action	Principal muscles involved	Protocol
Upper limb: Hyperextension or scapular retraction, forearm supination, elbow flexion, shoulder abduction, finger and wrist flexion	Upper limb: Pectoralis major and minor, brachioradialis, biceps brachii, latissimus dorsi, palmaris longus, flexor digitorum superficialis, profundas, and supinator	Position of the patient: sitting and supine
Trunk: pelvic retraction	Trunk: thoracolumbar fascia	Position of the therapist: Standing-stretching approaches are slow repetition, continuous stretching, and manual stretching
Lower limb: knee flexion, ankle dorsiflexion, inversion, hip flexion, abduction, and external rotation	Lower limb: Piriformis, rectus femoris, iliacus sartarius, psoas major, gluteus minimus, gluteus medius, hamstring, and tibialis anterior	Doses: 3 sets, held for 30 seconds each

Investigations

Physiotherapy Intervention

The physiotherapy intervention was given for six weeks, five days a week. The patient received mCIMT in addition to conventional physical therapy.

Modified CIMT: Exercises were focused on the paretic lower extremities. These movements were a part of the 30-minute MCIMT strategy, including side stepping, stair climbing, ball kicking, knee control on a step, and transfer package (10 minutes each session).

Conventional therapy: Exercises include stretching the flexor synergistic (as shown in Table 5), strengthening exercises using a theraband (shown in Table 6), reaching beyond arm's length that is practiced in sitting and standing postures as a part of balance training, training for walking that involves challenge to dynamic balance (such as obstacle courses), and range of motion in the upper and lower extremities (passive and active assisted).

**Table 5:** Upper extremity, pelvic position, and lower extremity strengthening exercises

Muscle action	Principal muscles involved	Protocol
Upper limb: Forearm supination, elbow flexion, shoulder abduction, scapular retraction or hyperextension, finger and wrist flexion	Upper limb: Triceps, pronator teres, latissimus dorsi muscle, deltoid, extensor digiti minimi, and extensor digitorium	Position of the patient: Sitting and supine
Trunk: Pelvic retraction	Trunk: Quadratus lumborum	Position of the therapist: Standing
Lower limb: Knee flexion, ankle dorsiflexion, inversion, hip flexion, abduction, and external rotation	Lower limb: Gracillis, gluteus maximus, quadriceps, gastrocnemius, adductor mangus, adductor brevis, and adductor longus	Progression: Using a theraband (colored yellow, red, and green), strengthening exercises will be performed.

**Table 6:** Outcome measures

	Pre-rehabilitation	Post-rehabilitation
Berg balance scale	07/56	52/56
Dynamic gait index	04/24	22/24
Trunk impairment scale	11/23	22/23
Functional reach test	3 inch	10 inch
10-meter walk test	4 meters with a walker	10 meters without a walker
Fall efficacy test	26/36	10/36

## Discussion

With aging comes an increased risk of stroke because of concomitant conditions, such as atrial fibrillation, ischemic heart disease, and hypertension. Out of 50% of patients, 56% had left hemiplegia, 42% had right hemiplegia, and 2% had bilateral hemiplegia. In most cases, left-sided infarcts in MRIs are easier to identify than right-sided ones [6]. The outcome measure score has been improved. An evidence-based study supporting this conclusion found that early and more intense physical therapy improves motor functions and the capacity to carry out everyday tasks after a stroke [10].

In the majority of the cases, mCIMT showed improvement in motor functions. Several researchers have used a combination of treatments that have shown to be quite advantageous. Corbetta et al. concluded that CIMT is a multimodal strategy that combines increased activity that is appropriate for one's capacity with minimizing the usage of the non-paretic extremity. Research revealed that while there were modest gains in motor impairment and motor function related to CIMT, these advantages did not significantly lessen disability. The outcome of our earlier meta-analysis suggested that CIMT would be better than conventional rehabilitation [11]. Candan et al. concluded that compared to neurodevelopment treatment (NDT), in stroke patients, mCIMT for paretic lower limbs outperformed NDT in improving motor function (gait parameters, balance, ambulation, and symmetry). For the rehabilitation of the lower limbs, mCIMT may be a novel alternative treatment [12]. According to the study's findings, the center of mass sagittal plane displacement increased considerably during mCIMT in comparison to conventional therapy, and the benefits of mCIMT on stroke patient’s hemiplegic gait may include improved lower limb driving force, decreased energy expenditure, and improved balance [13].

Numerous research works have shown that mCIMT is beneficial in enhancing upper limb function. In stroke patients, mCIMT improved functional mobility, decreased test execution time, and increased gait metrics. In this work, we demonstrate the effectiveness of mCIMT in improving lower limb function by addressing the "learned non-use" principle for gait parameters and balance and by using three fundamental elements, namely, the use of repetitive, structured, practice-intensive therapy in the paretic limb and constraint of the non-paretic limb.

## Conclusions

According to the studies, mCIMT, which is mostly applied to the upper limbs, involves using the paretic limbs while restricting the use of non-paretic limbs. However, recent studies have shown that the treatment can be used to help lower limbs retain balance and improve gait. Patients who have experienced a stroke typically struggle with ADLs because of weakness in their paretic limbs; for this reason, paretic limb rehabilitation has been proven to be beneficial. This case report shows that the patient achieved his maximum goal by using mCIMT, and the patient also achieved normalized tone, better sitting and standing balance, improved range of motion, and muscle strength on the affected side. mCIMT is beneficial in improving motor control, quality of life, and independence in ADLs. Hence, we conclude that mCIMT is a promising strategy for the rehabilitation of stroke patients, in addition to primary rehabilitation.
